# Clinical and molecular fingerprint of SARS-CoV-2 among hospital employees in a period of Omicron BA.2 dominance

**DOI:** 10.3205/dgkh000531

**Published:** 2025-02-24

**Authors:** Dominic Rauschning, Ruth Weppler, Carsten Balczun, Gwendolyn Scheumann, Jasmin Monteiro Marques, Christina Mutschnik, Dominic Preuß, Ricarda Maria Schmithausen, Maximilian Starke, Ralf Matthias Hagen, Manuel Döhla

**Affiliations:** 1 Bundeswehr Central Hospital Koblenz, Department I B: Internal Medicine, Koblenz, Germany; 2 University Hospital Cologne, Department I of Internal Medicine, Cologne, Germany; 3 Bundeswehr Central Hospital Koblenz, Department XXI: Microbiology and Hospital Hygiene, Koblenz, Germany; 4 Bundeswehr Central Hospital Koblenz, Department XVI: Laboratory Medicine, Koblenz, Germany; 5 Bundeswehr Central Hospital Koblenz, Department XXIV: Hospital Pharmacy, Koblenz, Germany; 6 Institute of Hygiene and Public Health, University Hospital, Medical Faculty, University of Bonn, Germany; 7 Bundeswehr Central Hospital Koblenz, Department II: Visceral Surgery, Koblenz, Germany; 8 Bundeswehr Central Hospital Koblenz, Department III: Dermatology, Koblenz, Germany

**Keywords:** SARS-CoV-2, COVID-19, Omicron BA.2, COVID-19 symptoms, testing strategy, contact testing, clearance testing, ECLIA, antigen testing, hospital hygiene

## Abstract

In the spring of 2022, SARS-CoV-2 Omicron BA.2 peaked in Germany. The main burden was staff shortage. To achieve effective identification and management of infected persons as well as early reintegration of recovered persons, an infection-control outpatient clinic was established at the Bundeswehr Central Hospital Koblenz. This article reports a secondary data analysis of 663 people with 1,174 visits to the outpatient clinic. For asymptomatic contacts, no correlation was observed between PCR result and testing time or frequency. Although no significant symptoms were documented, a high correlation was found between a positive antigen self-test and positive PCR. For clearance, a median time until a negative test was obtained was 8–11 days. The PCR gold standard was compared with ECLIA antigen testing for all indications. The results of this study challenge the rationale for testing asymptomatic contacts. Solely symptom-driven diagnostics by PCR also do not seem to be effective. However, contact persons or symptomatic persons with a positive rapid antigen test should be tested further. Whether this testing is done by ECLIA or PCR does not seem to matter. Clearance testing after recovery prior to day 8 is also not appropriate.

## Abstract (de)

Im Frühjahr 2022 erreichte SARS-CoV-2 Omikron BA.2 in Deutschland seinen Höhepunkt. Die Hauptbelastung entstand durch Personalmangel. Um eine effektive Identifizierung und Behandlung von Infizierten sowie eine frühzeitige Wiedereingliederung von Erkrankten zu erreichen, wurde am Bundeswehrzentralkrankenhaus Koblenz eine Infektionsschutzambulanz eingerichtet. In diesem Artikel führen wir eine Sekundärdatenanalyse von 663 Personen mit 1.174 Besuchen in der Ambulanz durch. Bei asymptomatischen Kontakten konnte keine Korrelation zwischen PCR-Ergebnis und Testzeit oder -häufigkeit festgestellt werden. Wir konnten keine signifikanten Symptome feststellen, aber eine hohe Korrelation einem positiven Antigen-Selbsttest und einer positiven PCR. Für die Freitestung wurde eine mittlere Zeitspanne von 8–11 d bis zu einem negativen Test ermittelt. Wir haben den PCR-Goldstandard mit dem ECLIA-Antigentest für alle Indikationen verglichen. Unsere Ergebnisse stellen die Rationale für die Testung asymptomatischer Kontaktpersonen in Frage. Eine rein symptomorientierte Diagnostik mittels PCR scheint ebenfalls nicht effizient zu sein. Kontaktpersonen oder symptomatische Personen mit einem positiven Antigen-Schnelltest sollten jedoch weiter getestet werden. Ob diese Tests mittels ECLIA oder PCR durchgeführt werden, scheint keine Rolle zu spielen. Freitestung nach der Genesung vor dem 8. Tag sind ebenfalls nicht angebracht.

## Introduction

The omicron variant of SARS-CoV-2, with its milder courses but higher infectiousness, led to extensive staff shortages in almost all economic sectors due to government regulations on mandatory isolation [1], [2]. In Germany, the BA.2 variant caused high incidences in the spring and early summer of 2022 [3]. The health system in particular was hit hard, which made it much more difficult to deal with the pandemic [4]

To limit absences, appropriate testing strategies and diagnostics are needed that can guarantee a reliable negative test result.

The most preferred test for validly diagnosing a SARS-CoV-2 infection is reverse transcriptase quantitative PCR (RT-qPCR), although weaknesses have been shown [5], [6]. This method usually offers high sensitivity and specificity and allows determination of the underlying virus variant [7], [8]. However, it is resource-intensive and requires extensive material equipment and qualified personnel, while the electrochemical luminescence immunoassay (ECLIA) method offers the possibility of examining large sample quantities in a largely automated process. A result can be obtained within 30 min [9].

This retrospective observational study at a German military hospital had two aims:

- Evaluation of the in-house test strategy and
- comparison of the performance of ECLIA and PCR to draw conclusions for a refined test strategy for future waves of SARS-CoV-2 or other epidemic respiratory infections.

In the military context, infection surveillance and fast, simple, and readily available diagnostics play a decisive role in ensuring the operational readiness of the armed forces.

## Materials and methods

### Infrastructure and process organisation

On 21 March 2022, an infection-control outpatient clinic for the hospital’s staff was opened, operating Mondays through Fridays (excluding holidays).

All persons filled out a questionnaire regarding personal data, including rank for military personnel, vaccination and recovery status at their first visit. This questionnaire recorded the symptoms via checkboxes and free-text fields, results of any antigen self-tests, information on (infected) contact persons and pre-existing SARS-CoV-2 infection.

All persons consented to their data being collected and stored as well as analyzed anonymously in the future.

Three indication groups were categorized as defined by the Robert Koch Institute (RKI) [10]:

1. “Suspicious cases”: people with or without contact to a confirmed case presenting because of symptoms compatible with Covid-19 or because of a positive antigen self-test.
2. “Contacts”: people without symptoms or positive antigen self-test with contact to a confirmed case within 14 days before the visit. The regular testing scheme was day 1, 7, and 14 after contact.
3. “Clearance”: people could return to work after ending their officially ordered home isolation. The regular testing scheme was day 7 after a positive test, with at least 48 h without symptoms before the test.

In reality, there was a great deal of overlap between the groups “contacts” and “clearance”, for instance, depending on weekends, symptom progress or the date the contact status became known. Since 2 May 2022, a new testing scheme has been published, setting day 5 after a positive test as the first possible day for clearance testing.

All tests were carried out with one single swab for both their throat and both nostrils. We used MicroTest M4RT Universal Swab Kits (Thermo Fischer Scientific, Waltham, Massachusetts, USA). As these kits contain 3-ml vials of medium, it was possible to use every specimen for multiple analyses. The specimens were transported to the laboratory without any relevant delay and stored at 2–8°C.

### Laboratory analysis

For laboratory analysis, a two-part testing approach was employed. First, antigen testing (ECLIA) was performed. Second, the same specimen (inoculated medium) was used for PCR.

ECLIA was processed by Elecsys SARS-CoV-2 antigen assay against SARS-CoV-2 N protein (Roche Diagnostics GmbH, Mannheim, Germany) on the immunoassay analyzer platform Roche cobas e 801. The medium, inoculated with naso-/oropharyngeal specimens, was used at a ratio of 10:1 with SARS-CoV-2 Extraction Solution C (Roche Diagnostics GmbH, Mannheim, Germany) (500 µl specimen, 50 µl extraction solution). The test result was declared as reactive when the cut-off index was ≥1 (COI; comparison of electrochemiluminescence signal of the specimen with the cut-off as determined by calibration).

Due to the hospital’s process organization, PCR was performed either in the Department of Microbiology and Hospital Hygiene or in the Department of Laboratory Medicine. Both used different PCR platforms. In the Department of Microbiology and Hospital Hygiene, Laboratory-based RT-qPCR analysis for SARS-CoV-2 infection was carried out on a STARlet IVD platform for nucleic acids extraction (Seegene, Seoul, South Korea) and a CFX96 Dx PCR cycler (Bio-Rad, Hercules, California, USA) using the Allplex SARS-CoV-2/FluA/FluB/RSV assay (Seegene) as recommended by the manufacturer. In the Department of Laboratory Medicine, multiplex rapid real-time reverse transcriptase PCR testing was processed by Xpert^®^ Xpress CoV-2/Flu/RSV plus (Cepheid, Sunnyvale, CA, USA) on the platform Cepheid GeneXpert Infinity. The test detects targets in the SARS-CoV-2 genes RdRP, E and N2, but only provides a pooled result. 

### Statistical analysis

The data collected between 21 March and 22 May 2022, during the dominance of >85% SARS-CoV-2 Omicron BA.2 [3], [11], were analyzed, using STATA IC 15.1 (Stata Corp, College Station, Texas, USA) for statistical analysis. For the total collective (N), each person’s first visit to our clinic was used to describe age, sex, socioeconomic status (SES), vaccination status and recovery status. The categorical variables reported are absolute (n) and relative (%) proportions; for continuous variables, p25-value, median, p75-value and maximum are reported. The SES was classified according to the institutional pay grade, which takes into account formal qualification, activity and salary. For military personnel, it is based on rank, whereby enlisted soldiers were classified as “lower SES”, non-commissioned officers as “middle SES” and officers as “higher SES”. 

For all indication groups, sensitivity, specificity, PPV and NPV of the ECLIA technique compared to PCR were calculated and reported with exact binomial confidence intervals. Following the manufacturers’ and the RKI’s recommendations [10], results were defined as follows:

- A reactive ECLIA result was considered positive.
- A PCR result with ct values >0 in at least one examined gene was considered positive in “suspicious cases” and “contacts”.
- A PCR result with ct values of 0 or >30 in all examined genes was considered negative, with ct values >0 and ≤30 in at least one examined gene positive in “clearances”, because the RKI recommends viral loads beneath 10^6^ cop/ml be considered negative in this situation, due to assumed absence of infectivity. Since the correlation of viral load and ct value is laboratory- and device-dependent, the RKI recommends a ct value >30 as the cut-off value in practice.

The significance of a positive rapid antigen test was calculated dependent on a positive PCR result using 2x2 tables and an alpha level of 0.05 (2-sided) within each group.

**Figure 1:**
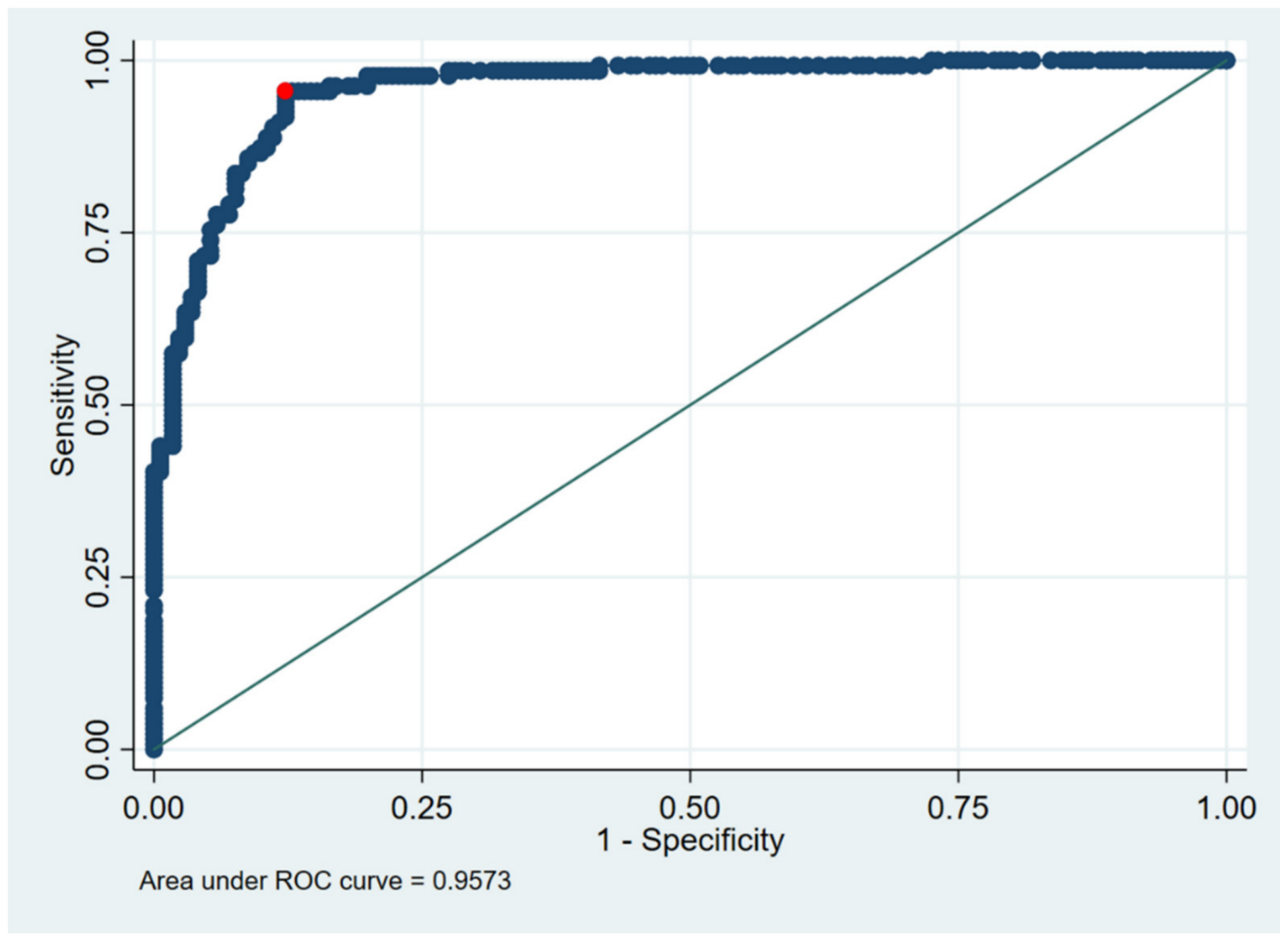
ROC analysis for ECLIA values in the clearance group (the best cut off – identical in Liu, Youden or nearest point method [12]) is 0.9505 (red dot); real cut off is 1.000).

In addition, further analyses were carried out in the different indication groups:

- Suspicious cases: The significance of reported symptoms was calculated dependent on a positive PCR result using 2x2 tables and an alpha level of 0.05 (2-sided).
  The symptoms were derived from the symptom checkboxes as well as the free text answers on the questionnaires, which were examined by a physician in case of ambiguity.
  For significant results, an odds ratio with 95% confidence interval was calculated.
- Contacts: The Odds Ratios for obtaining a positive test result in sequential testing after day 1–3, 4–9 and day 10 or later after contact were calculated and adjusted for age, sex, SES, vaccination and recovery state by a logistic regression model.
- Clearance: Based on the Youden, Liu and nearest point method [12], we calculated the optimal cut-off value for the clearance via ROC method (Figure 1 (Fig. 1)).

To determine the earliest day with a probably negative test after infection, Kaplan-Meyer plots were generated for each testing method (PCR, ECLIA, ECLIA with optimized cut-off). The earliest day was defined as the day after infection when at least 50% of the tests would react negatively, as well as the latest day after infection when at least 50% of the tests would still react positively. The difference between the two plots was defined as the median time span to earliest clearance. 

## Results

663 individuals (57.77% females) with a median age of 33.2 years (range 16.6–64.6) were examined. A large proportion (85.37%) had already received a first booster vaccination. The median number of visits per person was one (range 1–6), resulting in 1,174 visits in total. Table 1 (Tab. 1) gives an overview of the demographic data of the sample.

In detail, 423 visits were made due to contact alone, 371 due to suspicion, and 380 for clearance. In the contact indication, 33 of 422 evaluable test results were positive (7.8 %). In the group of suspicious cases, 178 of 367 evaluable tests were positive (48.5 %); 84 visits in this indication group had the status “contact person” (22.9%). However, they generated only 32 of the 178 positive tests (18.0 %). The clearance indication showed 105 positives of 380 evaluable tests (27.6 %). 

Suspicious cases mainly reported sore throat (n=156, 42.4%), rhinitis (n=147, 39.9%), cough (n=145, 39.4%), and headache and body pain (n=129, 35.1%) (Table 2 (Tab. 2)). A median of at least one symptom was reported (range 0–6). A significant association with a positive PCR result could not be demonstrated for any of the symptoms. Gastrointestinal symptoms spoke significantly against a positive PCR result (p=0.006). There was a significant correlation between reported symptoms (at least one reported symptom) or a positive antigen self-test and a subsequent positive result in PCR (each p<0.000) (Table 2 (Tab. 2)).

Examining the risk of obtaining a positive result as an asymptomatic contact person in a sequence of tests, a stable risk was observed during days 5 to 9 after the first test (OR 1.10, p=0.845) and a decrease when tested after day 10 (OR 0.44; p=0.296). However, these results are not statistically significant. 

**Figure 2:**
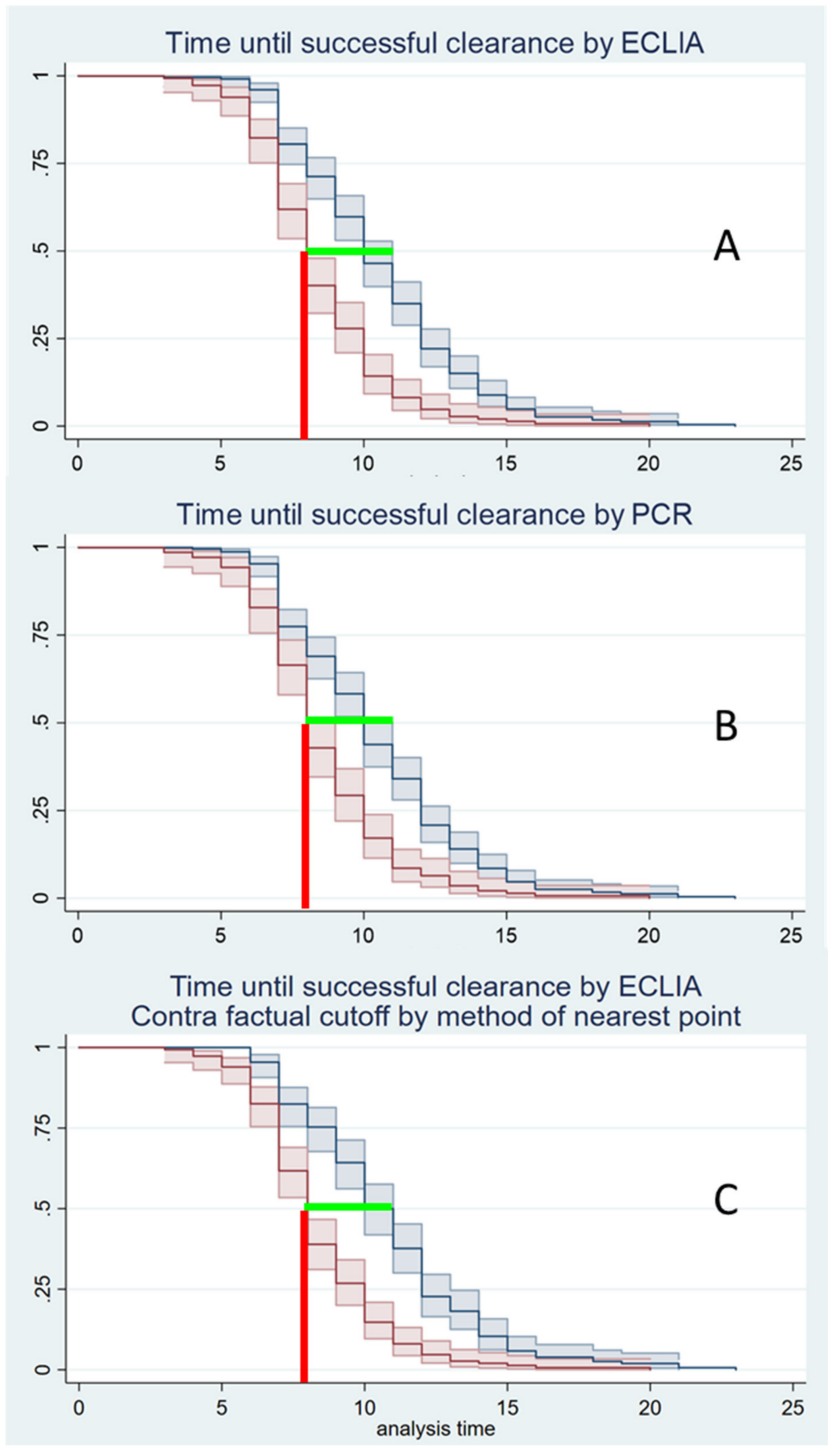
Estimated median time until successful clearance based on Kaplan-Meier curves. Red: estimator for 50% positive tests; blue: estimator for 50% negative tests; light red and light blue: 95% confidence intervals; green lines: span for the estimators in case of ECLIA (A) and PCR (B); contra-factual modelling of ECLIA cut-offs by nearest point to 0.1 (C); red lines: lower limit for the estimated median, which is identical (8 days) in all three variants.

The ROC analyses of the ECLIA testing showed the same optimal cut-off for all three statistical methods (Youden, Liu, nearest point): while the real cut-off was 1.0000, the optimal cut-off was 0.9505 (Figure 1 (Fig. 1)). However, there was no difference in the median time span to clearance using the optimized cut-off (Figure 2 (Fig. 2)).

Youden, Liu and nearest point method: The median time to clear the virus was 8–11 days. The earliest day on which a negative test result could be expected was the eighth day after first virus detection (Figure 2 (Fig. 2)) for both PCR and ECLIA. 

In addition, the ECLIA test procedure was analyzed in comparison to PCR. Suspicious cases with symptoms and/or a positive antigen rapid test showed a sensitivity of 88.6% and a specificity of 98.4%. The PPV was 98.1%, and the NPV was 90.3 % (Table 3 (Tab. 3)). 

Contact persons without symptoms and without a positive antigen rapid test showed a sensitivity of the test of 36.4% and a specificity of 99.5%. The positive predictive value (PPV) was 85.7%; the negative predictive value (NPV) was 94.9% (Table 4 (Tab. 4)). 

Finally, we examined the results of the clearance testing of both methods. The ECLIA had a sensitivity of 89.5% and a specificity of 80.5%. The PPV was 64.0%, the NPV was 95.2% (Table 5 (Tab. 5)).

## Discussion

The present results show that the clinical diagnosis of a SARS-CoV-2 infection is not possible in mild courses due to the lack of cardinal symptoms. While in previous variants, at least the loss of olfaction or taste was indicative [13], this study could not identify such a symptom. This is consistent with previously published results [14].

Notably, a negative correlation was observed between presenting with any symptoms and a positive test result. One explanation may be other respiratory viruses, which possibly led to increased symptomatic infections, as most of the pandemic measures in Germany (mandatory masks, social restraints, lockdowns, vaccine status-controlled access controls) had been weakened during the observed period. In line with this, the RKI reported a significant increase in respiratory non-COVID-19 infections during the spring and early summer of 2022 [15]. This could have caused symptoms of different viruses to overlap, so that no specific symptom for a specific pathogen was detectable. In our test battery, it was only possible to exclude RSV and influenza A and B.

Thus, it is all the more important that we were able to show a significant correlation between antigen self-test and PCR result. It was not possible to distinguish the reasons for which the self-test was performed by the employees in each case. But regardless of the particular reason, this study shows the importance of a self-test as a simple method of pre-selecting for more complex and expensive procedures such as PCR in order to reliably confirm an infection.

The present data did not provide any arguments for further contact-person testing. The risk of receiving a positive finding decreased, but not significantly, over time, especially after day 9. This fits with the pathophysiology and real-world data of omicron infection with a shorter incubation time of 3 days on average compared to previous variants [16], [17], [18], [19]. 

In this respect, regular testing of asymptomatic contacts over several days may have an unfavourable cost-benefit ratio. We conclude from this that ad-hoc testing without a cause (e.g., three times per week, as envisaged in the German Infection Protection Act [20] for hospitals, which was valid from October 2022 until April 2023) is even less useful. This explicitly does not mean that individuals cannot obtain a positive test result in individual cases. However, there is the question of whether these persons are relevant spreaders of SARS-CoV-2 at all [21], [22]. Moreover, the hygiene requirements in hospitals were still much stricter than the requirements in the public sector in Germany at the end of 2022 (for example FFP2/N95-masks during patient contact, medical face masks within all buildings of the hospital, increased attention and access to hand antisepsis). That means a nosocomial transmission of SARS-CoV was very unlikely, independent of compulsory testing of employees.

With regard to clearance testing, a median time of 8–11 days elapsed after initial detection before a negative test result was to be expected. Kojima et al. [23] showed a mean PCR positivity of 14.3 days at the beginning of the omicron phase in a cohort similar to ours in terms of sex and age structure. It remains to be seen to what extent the current German regulation of clearance testing is effective, recommending testing after 5 days if the patient has been symptom-free for 48 hours.

Comparing the PCR and ECLIA methods, different results were found for the three indication groups. These results are consistent with other studies which showed that the ECLIA can very reliably detect infections with a high viral load, but may have weaknesses in terms of sensitivity in terms of ct values >25–28 [24], [25], [26].

For the diagnosis of suspected cases, ECLIA showed a PPV of 98.1% and an NPV of 94.9% in our study population, thus providing a fully valid alternative to PCR. A false-negative result could be countered in the case of persistent clinical suspicion within the framework of a serial diagnosis (step 1 ECLIA for rapid infection control, step 2 multiplex-PCR for SARS-CoV-2, influenza A/B virus, RSV and possibly other respiratory viruses), or could be compensated by a test repetition in the case of a possibly higher viral load over time. Here, too, the hospital environment poses only an extremely low risk of transmission thanks to an appropriate hygiene concept. In addition, regardless of the diagnosis, sick persons should stay home at least until the end of the symptoms.

Notwithstanding our argumentation against testing of asymptomatic contacts, if one wants to perform regular asymptomatic testing, the higher sensitivity of PCR is certainly advantageous. On the other hand, PPV and NPV of ECLIA in this indication group were 85.7% and 94.9%, respectively. Considering the time to result, the cost of analytics, and the effect of repetitive testing, the efficiency of ECLIA might be equivalent or even superior. Further research on this aspect would be worthwhile.

For clearance, ECLIA also shows poorer values than PCR. However, as shown in Figure 1 (Fig. 1), this has no effect on the timing of a successful clearance, which is 8–11 days in both methods. This is surprising, since a ct >30 is “artificially” considered negative in PCR, whereas an ECLIA may still be positive in this case. Optimization of the cut-off, which was intended to counteract this factor, did not result in any change (Figure 1 (Fig. 1)). 

In summary, ECLIA represents a conceivable alternative for all three indications, particularly a speedy clearance, since ECLIA’s time to result (approx. 30 minutes) is much shorter than that of PCR (approx. 24 hours).

The results of the present study may not be applicable to the general population, nor to diagnostic testing of inpatients with more severe courses and possibly higher viral loads. Likewise, it is important to emphasize, especially with regard to leading symptoms and duration until clearance, that the present data refer to SARS-CoV-2 Omicron BA.2, and might differ for other variants. Further studies in other hospitals or occupational health settings would be useful, especially to examine the cost-benefit ratio of ECLIA versus PCR.

## Conclusions

Currently (November 2024), Germany does not require medical facilities to have a testing strategy for SARS-CoV-2 for their employees. 

For a voluntary testing strategy, however, we conclude that a serial testing of suspicious cases (step one: ECLIA on SARS-CoV-2, if negative: PCR on Influenza A/B virus, RSV, other respiratory viruses based on local epidemiological data) provides the best balance between a quick initial result to control SARS-CoV-2 and an accurate result over the disease course, mainly for epidemiological surveillance. Even with a negative ECLIA and PCR, symptomatic persons should stay at home for at least 48 hours after symptoms are disappeared or have significantly receded. There is no indication for contact testing of asymptomatic persons if sufficient hospital hygiene management is in place; nevertheless, a testing scheme based on self-testing may be an option, since positive self-testing is an indication for suspicious-case testing. ECLIA is an appropriate method for clearance testing, but the conditions to ensure validity are that it be performed (i) 48 hours after showing symptoms and (ii) at least eight days after the first positive test.

## Notes

### Authors’ ORCID

- Dominic Rauschning: 0000-0001-9284-8778
- Gwendolyn Scheumann: 0009-0000-4470-346X
- Ricarda M. Schmithausen: 0000-0002-3736-8672
- Ralf M. Hagen: 0000-0003-4875-1519
- Manuel Döhla: 0000-0001-8029-5264

### Ethical approval

Following the German guideline “Good practice secondary data analysis” (https://www.dgepi.de/assets/Leitlinien-und-Empfehlungen/GPS_revision2-final_august2014.pdf), a consultation with an ethics committee is not required for analyses based solely on secondary data.

### Funding

None.

### Acknowledgements 

We would like to thank Dagmar Wegner, PhD, B.Sc. for checking and improving the linguistic style.

### Competing interests

The authors declare that they have no competing interests.

## Figures and Tables

**Table 1:**
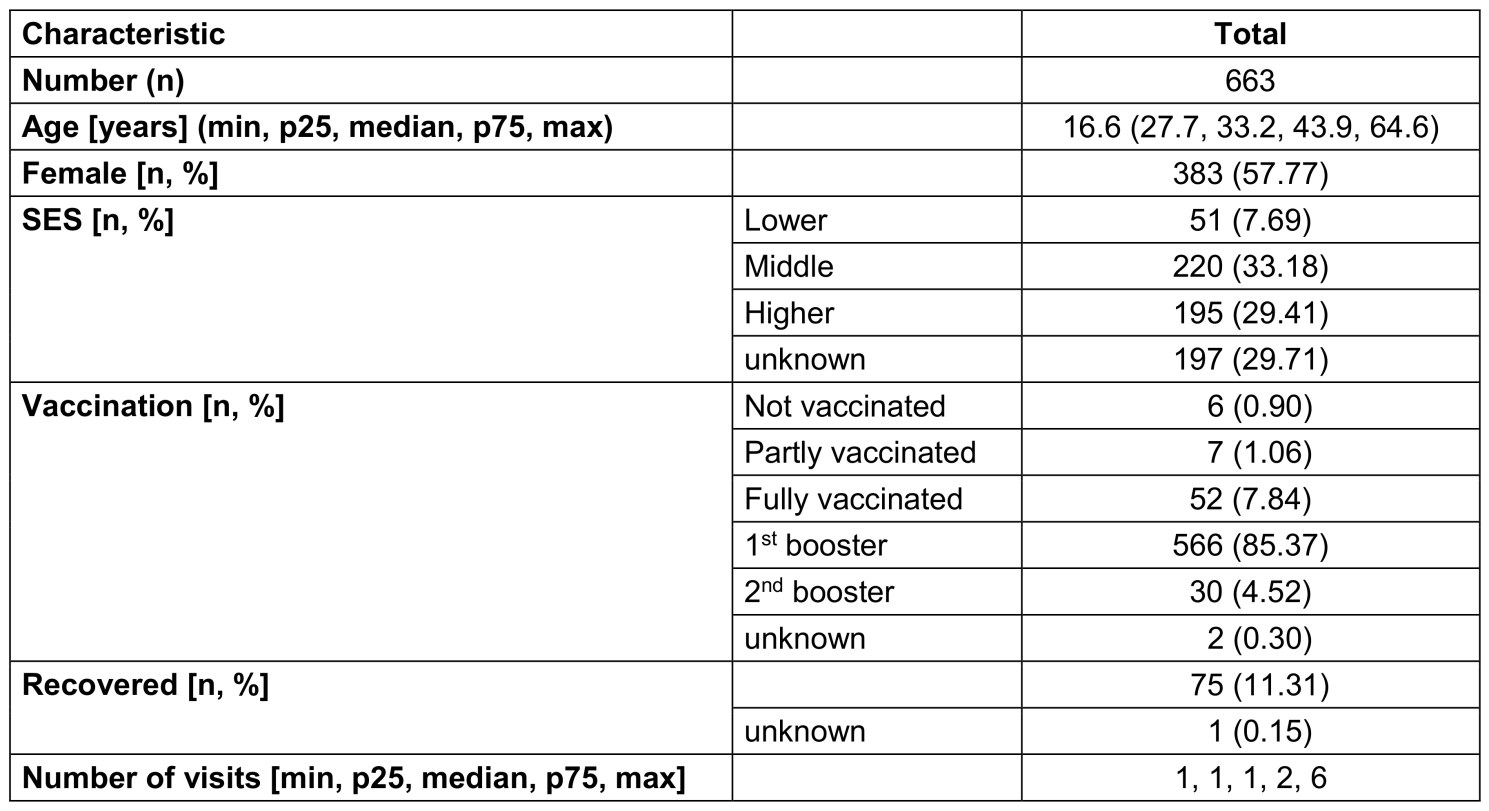
Description of examined staff (whenever a person visited the outpatient clinic more than once, only the first visit was counted for demographic analysis)

**Table 2:**
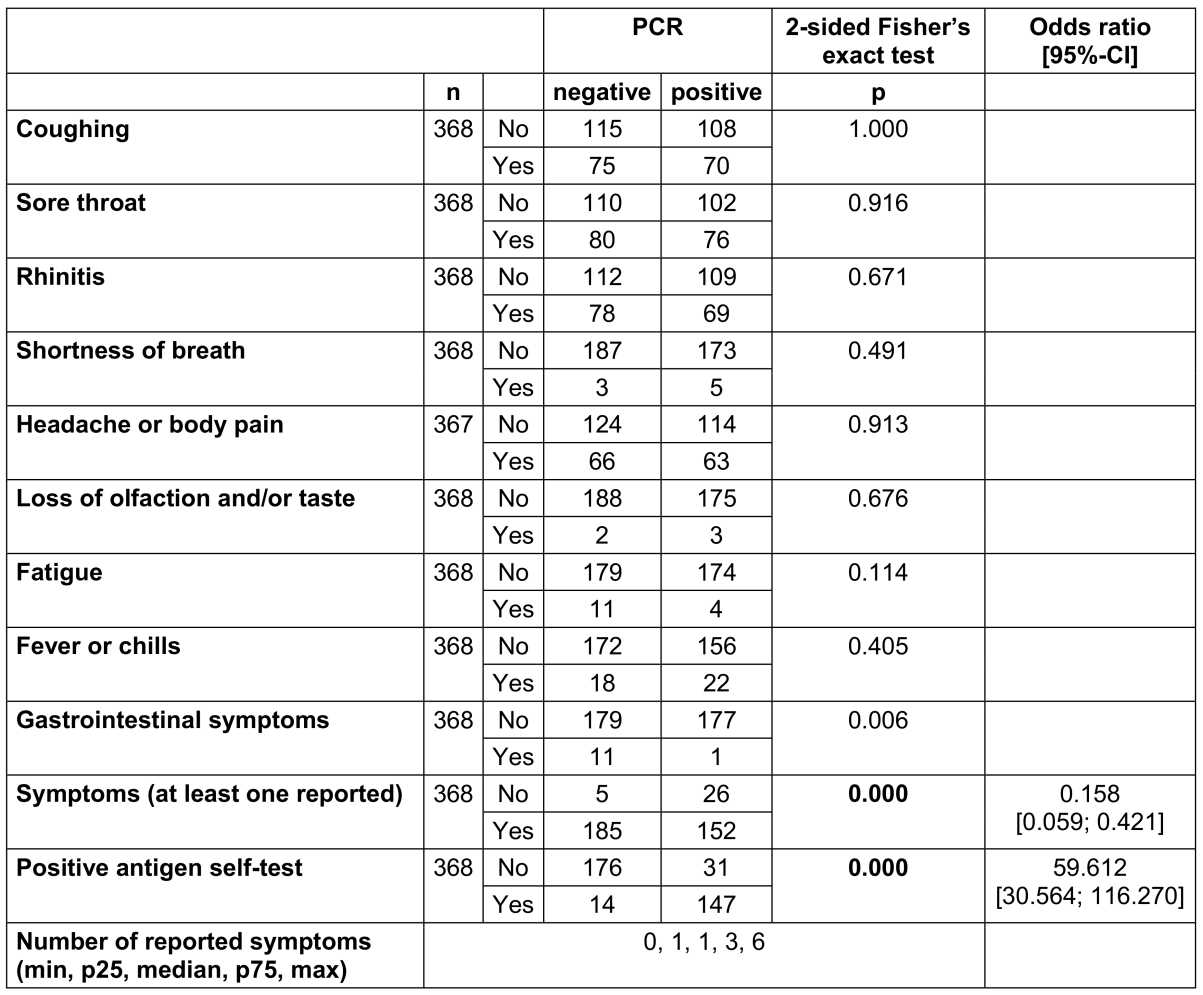
2x2 tables for observed symptoms and positive antigen self-test results in relation to the PCR result for visits of suspicious cases (whenever a person visited the outpatient clinic more than once, all symptoms ever mentioned were counted for analysis. Significant p-values are written in bold; n=371. n=number of evaluable results excluding missing results)

**Table 3:**
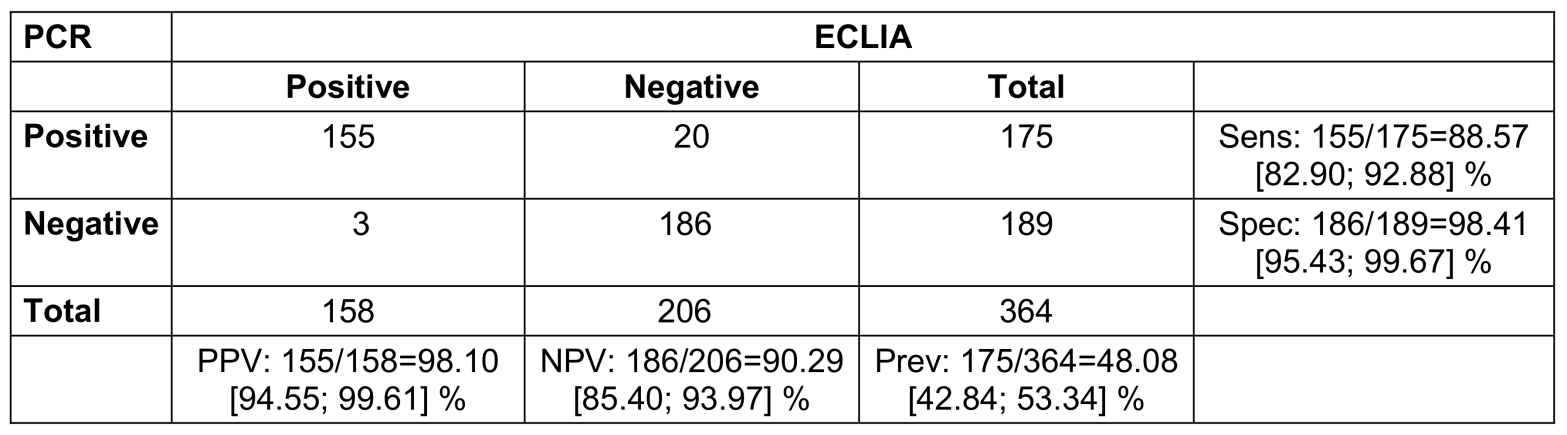
Suspicious cases with either symptoms and/or positive rapid antigen test, with or without contact person status (2x2 table for comparison of PCR and ECLIA with calculation of positive predictive value (PPV), negative predictive value (NPV), sensitivity (sens), specificity (spec) and prevalence (prev); square brackets give the 95% confidence intervals; n=371, 7 presentations had missing PCR results due to loss of sample.)

**Table 4:**
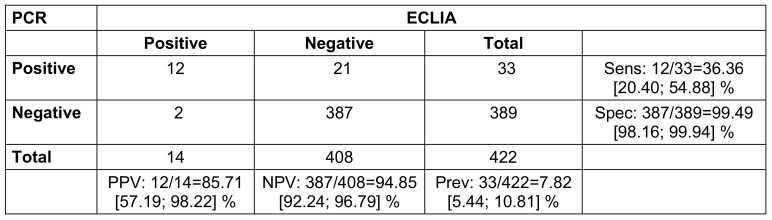
Contact persons without symptoms or positive rapid antigen test (2x2 table for comparison of PCR and ECLIA with calculation of positive predictive value (PPV), negative predictive value (NPV), sensitivity (sens), specificity (spec) and prevalence (prev); 95% confidence intervals in square brackets; n=423. One presentation had a missing PCR result due to loss of sample.)

**Table 5:**
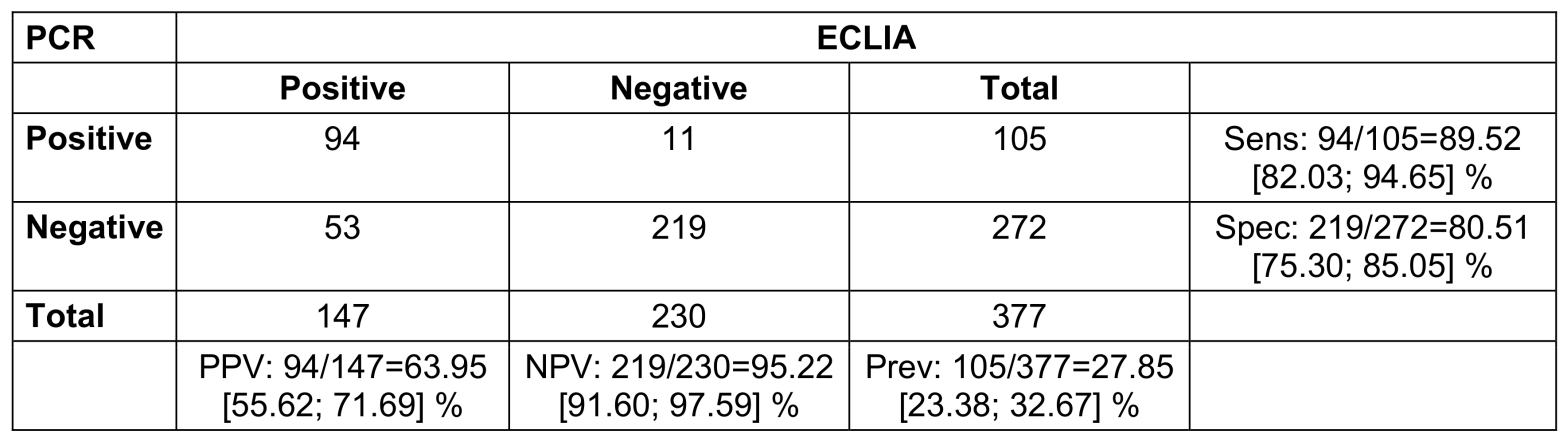
Clearance (real data, where PCR with at least one gene <30 counts as positive, all genes >30 or 0 count as negative; 2x2 table for comparison of PCR and ECLIA with calculation of positive predictive value (PPV), negative predictive value (NPV), sensitivity (sens), specificity (spec) and prevalence (prev); 95% confidence intervals in square brackets; n=423. One presentation had a missing PCR result due to loss of sample)
